# Prediction of Multiple Clinical Complications in Cancer Patients to Ensure Hospital Preparedness and Improved Cancer Care

**DOI:** 10.3390/ijerph20010526

**Published:** 2022-12-28

**Authors:** Regina Padmanabhan, Adel Elomri, Ruba Yasin Taha, Halima El Omri, Hesham Elsabah, Abdelfatteh El Omri

**Affiliations:** 1 Division of Engineering Management and Decision Sciences, College of Science and Engineering, Hamad Bin Khalifa University, Qatar Foundation, Doha 34110, Qatar; 2 Department of Hematology and Bone Marrow Transplant, National Center for Cancer Care and Research, Hamad Medical Corporation, Doha 3050, Qatar; 3 Surgical Research Section, Department of Surgery, Hamad Medical Corporation, Doha 3050, Qatar

**Keywords:** cancer care, public health, machine learning, febrile neutropenia, multidrug-resistant organism, hematological malignancies, Qatar

## Abstract

Reliable and rapid medical diagnosis is the cornerstone for improving the survival rate and quality of life of cancer patients. The problem of clinical decision-making pertaining to the management of patients with hematologic cancer is multifaceted and intricate due to the risk of therapy-induced myelosuppression, multiple infections, and febrile neutropenia (FN). Myelosuppression due to treatment increases the risk of sepsis and mortality in hematological cancer patients with febrile neutropenia. A high prevalence of multidrug-resistant organisms is also noted in such patients, which implies that these patients are left with limited or no-treatment options amidst severe health complications. Hence, early screening of patients for such organisms in their bodies is vital to enable hospital preparedness, curtail the spread to other weak patients in hospitals, and limit community outbreaks. Even though predictive models for sepsis and mortality exist, no model has been suggested for the prediction of multidrug-resistant organisms in hematological cancer patients with febrile neutropenia. Hence, for predicting three critical clinical complications, such as sepsis, the presence of multidrug-resistant organisms, and mortality, from the data available from medical records, we used 1166 febrile neutropenia episodes reported in 513 patients. The XGboost algorithm is suggested from 10-fold cross-validation on 6 candidate models. Other highlights are (1) a novel set of easily available features for the prediction of the aforementioned clinical complications and (2) the use of data augmentation methods and model-scoring-based hyperparameter tuning to address the problem of class disproportionality, a common challenge in medical datasets and often the reason behind poor event prediction rate of various predictive models reported so far. The proposed model depicts improved recall and AUC (area under the curve) for sepsis (recall = 98%, AUC = 0.85), multidrug-resistant organism (recall = 96%, AUC = 0.91), and mortality (recall = 86%, AUC = 0.88) prediction. Our results encourage the need to popularize artificial intelligence-based devices to support clinical decision-making.

## 1. Introduction

In 2020, 19.3 million new cancer cases were reported, as per GLOBOCAN 2020 statistics [1]. On the other hand, improvements in classical cancer care strategies, such as chemotherapy and radiotherapy, and the development of most modern treatment options, such as immunotherapy and targeted therapies (e.g., immune checkpoint inhibitors, monoclonal antibodies, and tyrosine kinase inhibitors), have improved the overall survival rate of cancer patients [2,3]. However, this increased survival rate also implies that there can be a significant impact on global health care due to the long-term cancer care demands of millions of cancer patients. The susceptibility of cancer patients to repeated and multiple infections is a leading factor that impairs the quality of life of cancer patients, increases hospitalization and healthcare burden, and poses yet another important threat due to the presence of multidrug-resistant organisms (MDRO) in their bodies. MDROs themselves pose a threat to global healthcare and, thus, one of the research priorities mentioned in the sustainable development goals (SDG) of the world health organization (WHO). Hematological cancer patients with acute lymphoid leukemia (ALL), acute myeloid leukemia (AML), lymphoma (LYM), and myelodysplastic syndromes (MDS) are at an increased risk of developing severe immune cell deficiency, which is the underlying cause behind the development of febrile neutropenia (FN). Such patients often report multiple infections, such as bloodstream infection (BSI), chest infections, sinus infections, colitis, urinary tract infection (UTI), and skin infection.

Common risk factors identified behind the incidence of FN in cancer patients include myelosuppression (secondary effect of chemotherapy), vitamin and iron deficiencies, age, genetic polymorphism, and other comorbidities, including autoimmune diseases. Compared to tumors, liquid cancers, which involve bone marrow dyscrasias, are at a higher risk of developing FN. Low immune cell counts during FN sometimes lead to multiple infections, which, in turn, trigger an overwhelming and fatal immune response (sepsis) along with impaired cardiac activity (septic shock) [4]. Septic shock (herein referred to as sepsis) related death accounts for 20% (48.9 million) of global deaths [5,6,7]. There is also evidence for a high frequency of inappropriate use of antibiotics leading to the development of MDRO and the inter-patient spread of MDROs in hospitals [8,9,10]. Early screening enables hospital staff to follow CDC-recommended standard precaution steps, such as using gloves, gowns, and high-standard sanitization measures while handling body secretions, excretions, ostomy tubes, catheters, needles, and any medical equipment that may come in close contact with patients with MDROs in their body [8,10,11]. Hence, prior identification of the presence of MDRO, susceptibility to sepsis, and mortality in hematological cancer patients with FN are imperative. Thus, the main objective of this paper is to propose machine learning (ML) models for the prediction of sepsis, the presence of MDRO, and mortality with available medical records toward recruiting specialists’ attention for managing high-risk patients. Another focus is to demonstrate the use of advanced ML strategies to deal with the issue of class imbalance in medical data. Data augmentation and specific model-tuning strategies are effective in this regard [12,13]. We also propose the use of a novel set of features and a comparatively lesser number of input variables for the prediction of three critical output variables.

The realm of medical data with contributions from electronic health records and results of clinical, molecular, and genomic experiments have fostered the development of artificial intelligence (Al)-driven medical decision support tools in various arenas of healthcare [14,15]. In parallel, AI-based computational models were suggested to predict the incidence of FN in solid and liquid cancers [16,17,18]. Apart from predicting FN incidence, such models brought into light the significant correlation of age, disease staging, treatment (e.g., taxane-based) regimen, and blood count taken 5 days after chemotherapy as features of high importance for predicting FN incidence [16,18]. As patients with FN are more likely to develop fatal complications, physicians often use clinical risk stratification indices based on the Multinational Association for Supportive Care in Cancer (MASCC) score and Talcott model for managing cancer patients with FN. These indices rely on the inpatient or outpatient status of the patient, the presence of comorbidities, and the aggressiveness of cancer to strategize patient care at the onset of FN. Similar to the prediction of FN incidence, a few AI-driven models were developed to predict patients with a risk of developing further FN-induced complications [19,20,21]. Specifically, in [21], the efficacy of an artificial neural network (ANN) model is evaluated against clinical scores, such as MASCC and Talcott. The ANN-based model predicted the outcome of chemotherapy-induced neutropenia in patients with solid (53.3%) and liquid cancers (lymphoma and leukemia) by using 34 clinical and laboratory parameters, including patient status, symptoms, treatment settings, use of antimicrobials and GCSF (Granulocyte colony-stimulating factor), cardiac measurements, fever duration, and complete blood count (CBC). The ANN model showed overall performance comparable to the clinically validated MASCC score in the Chinese population.

In [19], 226 features, including patient characteristics, clinical signs, details of therapy, and laboratory results, which were scaled down to 65 important ones, were used for the prediction of treatment outcomes of AML and LYM patients. Even though the random forest classifier displayed good AUC (75%), sensitivity (recall) was low (36%). While studies [19,20,21] looked at the risk stratification of patients into low and high-risk categories who are likely to develop further complications in general, others studied the applicability of AI-driven models for the prediction of one specific outcome, such as sepsis in children [22], bacterial sepsis in patients who received stem cell transplantation [6], the presence of bloodstream infection and transfer to pediatric intensive care [23], and mortality in adult FN patients [24].

An interesting XGboost-based ML model with high AUC (0.92) for predicting sepsis in children (<19 years old) who had BM transplantation is reported in [22], wherein the model required 23 features, including vital signs, complete blood count (CBC), coagulation function, serum CRP (C-reactive protein), electrolytes (ca, Na, K), liver function indicators, and infection index. One of the challenges for the implementation of the model discussed in [22] is the need for too many clinical measurements within 24 h of admission (at 4, 8, 12, and 24 h gaps), whose differential and aggregate values are used to derive features for model development. In [6], the model built for predicting bacterial sepsis and death in candidate patients for hematopoietic cell transplant showed AUC (0.85) and recall or sensitivity (80% (69–88)). Around 50 features collected during the initial 100 days from transplant, which included demographic, body structure, vitals and biomarkers, CBC, culture results, recent clinical events, donor relation, and disease type, were screened to investigate Staphylococcus aureus or Streptococcus bacteremia-lead death within 10 or 28 days of culture collection. In [24], 82 objective variables from administrative claims, which included ethnicity, Charleson’s comorbidity index, insurance type, household income, hospital stay, details of respiratory failure, renal failure, cardiac arrest, ventricular fibrillation, age, and other after care services, were used to predict mortality in cancer patients who present with FN. The linear support vector machine (LSVM), ridge-logistic regression (LR), gradient-boosting tree (GBT), and ANN models were built on selected objective administrative variables. Even though all four models displayed good precision (84%) and specificity (≥88%), the recall was 69%, 69%, 70%, and 69% for LSVM, Ridge-LR, GBT, and ANN, respectively. Despite the insights and clinical advantages promoted by the above models, their limitation in predicting the event of interest or rare clinical endpoint is a caveat. Hence, in this paper, we also demonstrate the use of model-tuning strategies and data augmentation methods to improve the prediction of the events (sepsis, MDRO, and mortality), in particular to overcome the low recall rate reported in the literature.

The organization of the paper is as follows: Section 2 is the methodology, including study population, data preprocessing, feature selection, model selection, and hyperparameter tuning of selected models for the prediction of sepsis, MDRO, and mortality. Section 3 discusses the results, particularly the robustness of the final predictive models derived using three parameter estimation strategies. Section 4 and Section 5 present the discussion and conclusions.

## 2. Methodology

### 2.1. Data and Study Population

Data are retrieved retrospectively from the medical database at the national center for cancer care and research (NCCCR), Qatar. The study population includes 513 patients with hematological cancer and FN who were admitted to NCCCR, Qatar, during the period 2009–2019, with 1166 episodes of FN characterized by fever (≥38.3${}^{\circ }$ and low levels of neutrophils (<500 cells/mL).

First, 16 relevant features were identified from the initial raw data retrieved from the medical record according to clinical advice and ease of availability, which included patient characteristics (age, gender, region (ethnicity), etc.), diagnostic category, disease status, type of infection, treatment phase, culture sensitivity for each of the 6 infections under study, and outcomes (sepsis, MDRO, mortality) (Table 1). MDRO is defined as a binary class derived from culture sensitivity records; this column marks patients with and without detected MDRO and is labeled as 1 and 0, respectively. The dataset included mainly four disease categories, namely acute lymphoid leukemia (ALL), acute myeloid leukemia (AML), lymphoma (LYM), and myelodysplastic syndromes (MDS). The types of infection included details of bloodstream infection (BSI), chest infection, sinus infection, colitis, urinary tract infection (UTI), skin infection, and whether BSI is line (catheter)-related or not. BSI infection is most prevalent (36.62%), and the main causative microorganisms were fungus, gram-negative (GN) bacteria, gram-positive (GP) bacteria, and their combinations.

All variables, except age, are categorical, and some have binary values (dichotomous variables yes = 1, no = 0) while some others, such as diagnostic category, are multiclass. Table 2 and Table 3 show the distribution of the continuous and dichotomous clinical characteristics of the patient cohort in the sepsis versus non-sepsis, MDRO versus non-MDRO, and mortal versus non-mortal groups, respectively. The variables chest infection, presence of multiple microbes in bloodstream (BSI polymicrobial), line-related infection, and urinary tract infection (UTI), in Table 2 are significantly higher in the sepsis group compared to the cohort that does not report sepsis (non-sepsis group), which indicates the discriminative ability of these features. Similar comparisons are given in Table 3 and Table 4 for MDRO and Mortality, respectively.

### 2.2. Preprocessing

**Figure 1:**
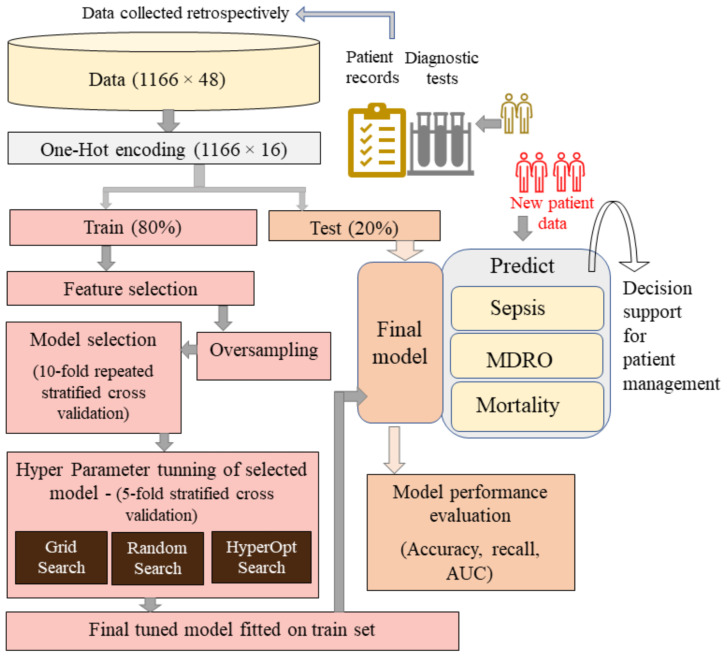
Schematic representation of the adopted methodology for building predictive models. The model is trained using 80% of the available data and tested using the rest of the held-out data (unseen by the model during training). When new patient data are available, the final model in the hospital decision support can be used for the prediction of sepsis, MDRO, and mortality for efficient patient management.

Figure 1 shows the data flow and various processing steps adopted for the development of the predictive model. As there are both nominal (e.g., region, disease type) and ordinal (e.g., stage) categorical variables in the dataset, one-hot encoding is used to create binary (0 or 1) columns with respect to each subclass under a multiclass categorical variable. The careful segregation of multiclass categories was performed to avoid the generation of sparse columns (very less frequent subclass) after one-hot encoding, which otherwise would lead to very deep and overfitted predictive models. As shown in Figure 1, after encoding, the dataset was split into 80% and 20% for training and testing, respectively. Note that for an imbalanced dataset, encoding before the data splitting step is desirable to avoid the issue of missing columns corresponding to certain subclass in the test set, leading to a smaller number of encoded columns and vector mismatch issues when presented to a model trained with all subclasses.

The correlation analysis conducted using 16 features in the training set revealed no perfect positive (>0.9) or highly positive (>0.5 or 0.7) attributes (See Figures S1 and S2 in the supplementary file). As the presence of MDRO in BSI has a higher correlation with the outcome compared to MDRO in all six infections, we used MDRO in BSI. The final feature set (predictive variables) with 12, 13, and 8 attributes for sepsis, MDRO, and mortality, respectively, were chosen based on the correlation analysis, forward feature selection using logistic regression, and inbuilt feature selection in XGboost (See Figures S6–S8 and Table S1 in the supplementary file). Table 2, Table 3 and Table 4 show the final features used for predicting sepsis, MDRO, and mortality, respectively.

**Figure 2:**
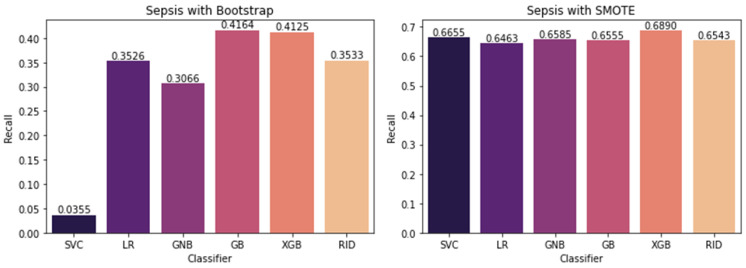
Comparison of various classifiers SVC: support vector machine, LR: logistic regression, GNB: Gaussian Naive Bayes, GB: gradient boosting, XGB: XGboost, and RID: ridge classifiers were used. Only the training set (80% of data) is used for data augmentation and model selection.

Apparently, learning to identify the minority class event (sepsis, MDRO, mortality) is more important than accurately predicting the majority class event (non-sepsis, non-MDRO, non-mortal). As given in Table 2, Table 3 and Table 4, for all three outcomes, the dataset is imbalanced wherein the ratio of the event to total instances is 229/1166, 215/1166, and 66/513 for sepsis, MDRO, and mortality, respectively. In medical datasets, data pertaining to events (sepsis, death, presence of MDRO) will be rare compared to the control set. Hence, the model gets a comparatively smaller chance to learn the rare events. However, it learns the likelihood of a patient being in the control class (class 0). This will create a bias in the model, wherein it can predict class 0 with high accuracy but fails to detect events (class 1). Data augmentation techniques allow us to up-sample the class 1 (minority) data set. Leveling up an imbalanced dataset is effective in avoiding ending up with a majority class-biased model after training, resulting in a high rate of misclassification, especially false negatives, which is critical. Data resampling, specifically oversampling methods, such as bootstrap and SMOTE (Synthetic Minority Oversampling Technique), can be used for handling data imbalance. The bootstrap method involves random data resampling until minority, and majority class balance is achieved where the model will see the same patient data multiple times. In SMOTE, new samples are generated from the existing minority class data by following the K-nearest neighbor method, wherein the algorithm selects a random nearest neighbor to create a synthetic instance in the feature space. As shown in Figure 2, we attempted to use both bootstrap and SMOTE methods of oversampling for conducting model selection. While the bootstrap method simply replicates the existing data, the SMOTE technique generates similar data points based on the existing minority points and provides more learning opportunities for the model while training. Thus, SMOTE increases the generalization capability of the predictive model, and hence improved performance is expected.

### 2.3. Model Selection

Initially, we considered six machine learning models, including support vector machine (SVC) with the Gaussian kernel, logistic regression (LR), Gaussian Naïve Bayes (GNB), gradient boost (GB), XGboost, and ridge classifier as candidate models. As shown in Figure 1, the 10-fold repeated stratified cross-validation strategy is used to compare different model’s expected performances on a dataset to facilitate the model selection. Figure 2 shows the average performance score exhibited by the model when k-1 folds of the training set were used for fitting and the kth fold of the training set was used for testing. Comparatively, the SMOTE method resulted in better model performance in all cases, with an average recall of 66%, whereas the average recall with the bootstrap method was 31%. With SMOTE, all the models exhibited comparable performance, with XGboost showing a slight upper hand. Hence, we conducted further experiments with the XGboost model, which combines the advantages of other tree-based models as well (random forest, decision trees). XGboost got its name due to the extreme gradient boosting strategy used by the algorithm to optimize the classifier toward robust decision-making. Moreover, it has parameters that can be particularly tuned to tackle issues with imbalanced datasets. In addition, XGboost has many advantages compared to other models; its fast convergence, simplified calculations to lower computational load, and inbuilt route to handle missing data in the dataset are some of them. It can be seen from Figure 2 that, even with XGboost, the recall achieved is 69% which needs to be improved. Hence, we used hyperparameter tuning.

### 2.4. Hyperparameter Tuning

As shown in the hyperparameter tuning block in Figure 1, we used three optimization procedures, grid search, random search, and hyperopt search (Bayesian optimization), to search for hyperparameters, which will minimize the cost function (error) toward obtaining the best model performance with respect to a given parameter search space. In the case of the grid search, a grid or matrix formed using preassigned values is used to choose a combination of hyperparameters. Whereas in the case of random search, optimal parameter values are evaluated over a bounded range of values in the search space. While grid search seeks the best parameter combination using every given value, random search selects only n random values in the given range of parameter space to identify the best parameter, where n is decided by the number of iterations (n_iter). Hence, choosing a sufficiently high number of iterations leading to the choice of a similar or higher number of parameters than the grid search may lead to the identification of better parameters. Otherwise, performance improvement is not guaranteed. As XGboost has several model parameters, hyperparameter tuning for the classifier is challenging and hence, intense and tedious. Due to this reason, exhaustive search methods for tuning the classifier will be a computationally costly option. Hyperopt, a type of Bayesian optimization (BO), is useful, especially for models with too many parameters. The BO wherein a probabilistic model (a surrogate function) is used to minimize the error is another suggested option as it requires fewer iterations than random search. This is achieved by implementing an informed way of learning based on the past evaluations conducted for hyperparameter searches [25]. Such learning enables it to ignore useless parameter space, allowing better search results in less time. In the case of hyperopt, based on the type of surrogate function used, there are different types of BOs, out of which we use Tree Parzen Estimator (TPE) as (1) it allows a variety of (e.g., uniform, quantized log-uniform, log-uniform, normally-distributed) search spaces and (2) it is good at handling categorical hyperparameters, wherein quniform (discrete) and uniform (continuous) parameters can be used to pass integers and floats spaced evenly, respectively. Optimization using the TPE algorithm is predicated on past results, and surrogate model evaluations are utilized to select the next set of hyperparameters, thus allowing maximum expected improvement.

Regarding the scoring method (metrics) to be used to evaluate the model tuning, the choice relies on the impact of true positives, true negatives, false positives, and false negatives with respect to the clinical endpoints, such as sepsis, MDRO, or mortality. In our case, we use the model to priorly predict high-risk patients to prioritize patients who need urgent attention. For instance, in the case of identifying patients with sepsis, patients who have sepsis and are predicted to have sepsis (true positive), and patients who have sepsis but are predicted as no-sepsis (false negative) is crucial; we need to maximize true positives and minimize false negatives. The other two categories are true negatives (no sepsis, predicted no sepsis) and false positives (no sepsis, predicted as sepsis). False positives are costly in terms of resources and initiating any psychological issues in a patient due to misdiagnose; however, not as detrimental as a false negative. An accuracy metric is recommended when true positive and true negative are important and recall (sensitivity) when the cost of false negative is high. Interestingly, with our dataset, in the case of sepsis, with model scoring metrics set to accuracy, the model can achieve over 70% accuracy without even predicting a single patient with sepsis correctly, but only by predicting all patients with no-sepsis accurately. Note that the test set is not oversampled and has disproportionate classes (70% no sepsis and rest with sepsis). Hyperparameter tuning with scoring metrics set as accuracy or AUC leads to a poorly performing model, especially in terms of minority prediction. Therefore, we use scoring = “recall” for grid search and random search and eval_metrics = “aucpr” for hyperopt while conducting hyperparameter tuning. Finally, as shown in Figure 1, we used accuracy, recall, and AUC for the final model assessment. The higher the AUC, the better the model is at predicting a no sepsis class as negative (0) and a sepsis class as 1. AUC reflects the classification capability of the classifier under varying discriminative thresholds.

**Figure 3:**
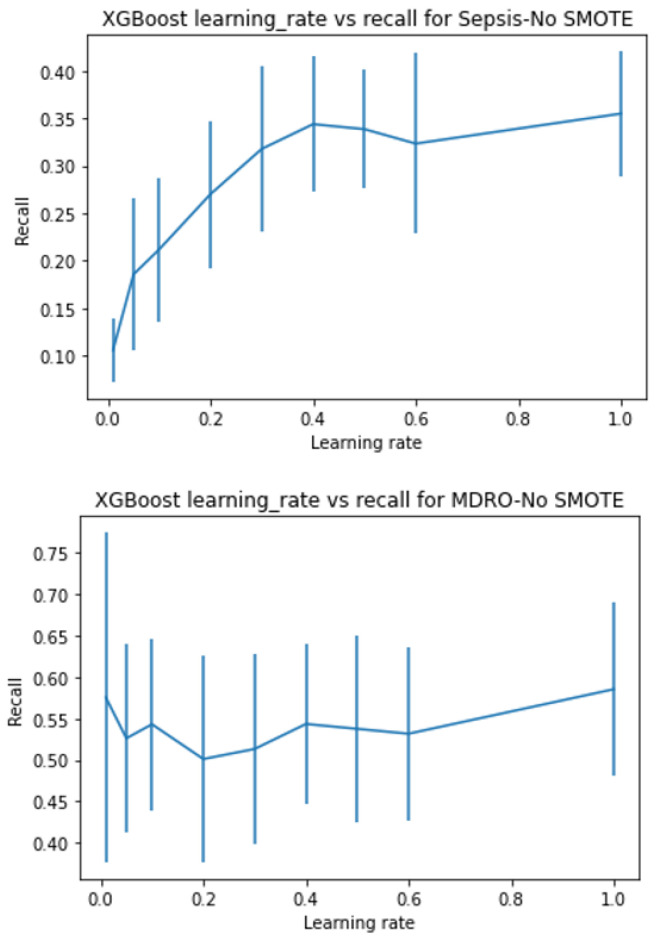

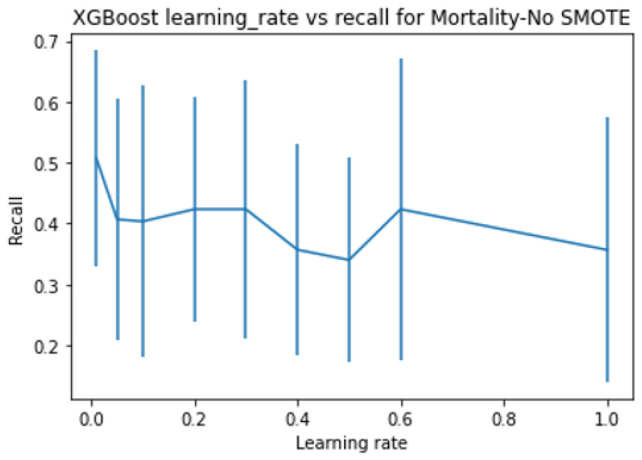
Shows the recall versus learning rate for all three outcomes without data augmentation on the training set, plotted using XGboost as the base model. Without data augmentation, the event prediction capability of the models are 10–35%, 50–57%, and 35–50% for sepsis, MDRO, and mortality, respectively.

**Figure 4:**
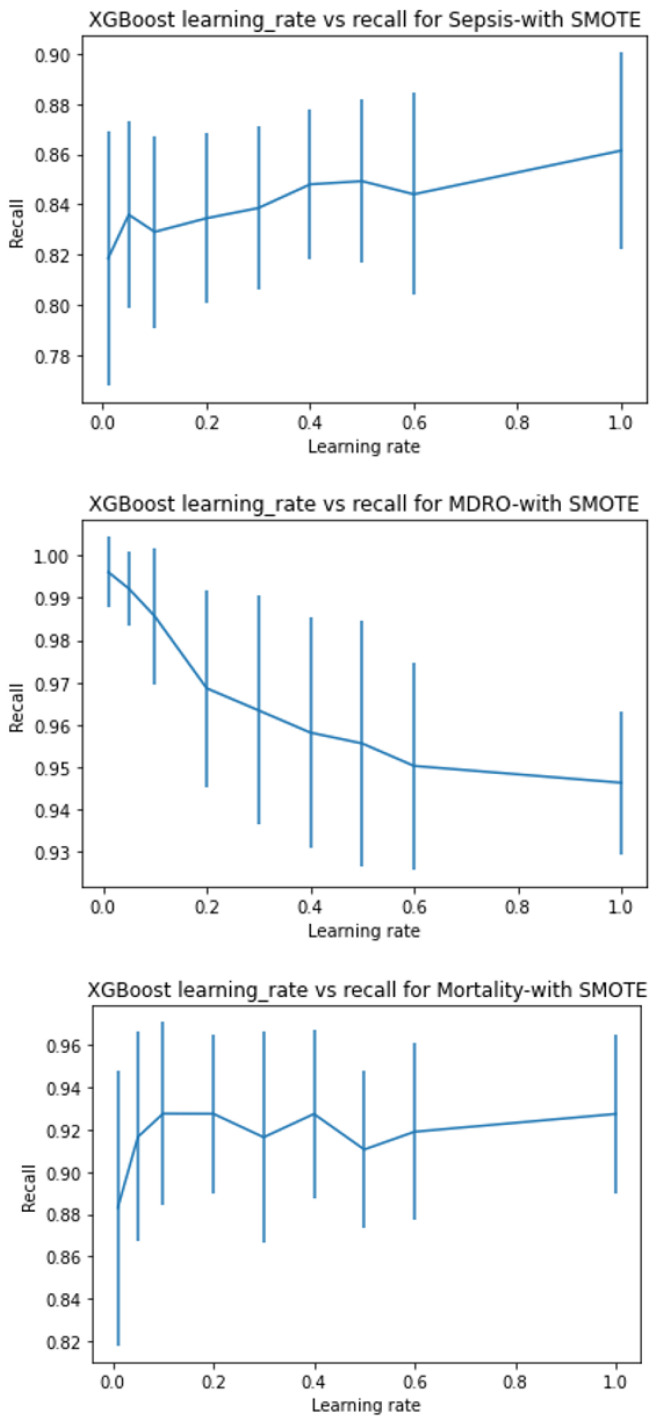
Shows the recall versus learning rate for all three outcomes with data augmentation on the training set, plotted using XGboost as the base model. With data augmentation, the event prediction capability of models are 82–85%, 95–99%, and 88–93% for sepsis, MDRO, and mortality, respectively.

**Figure 5:**
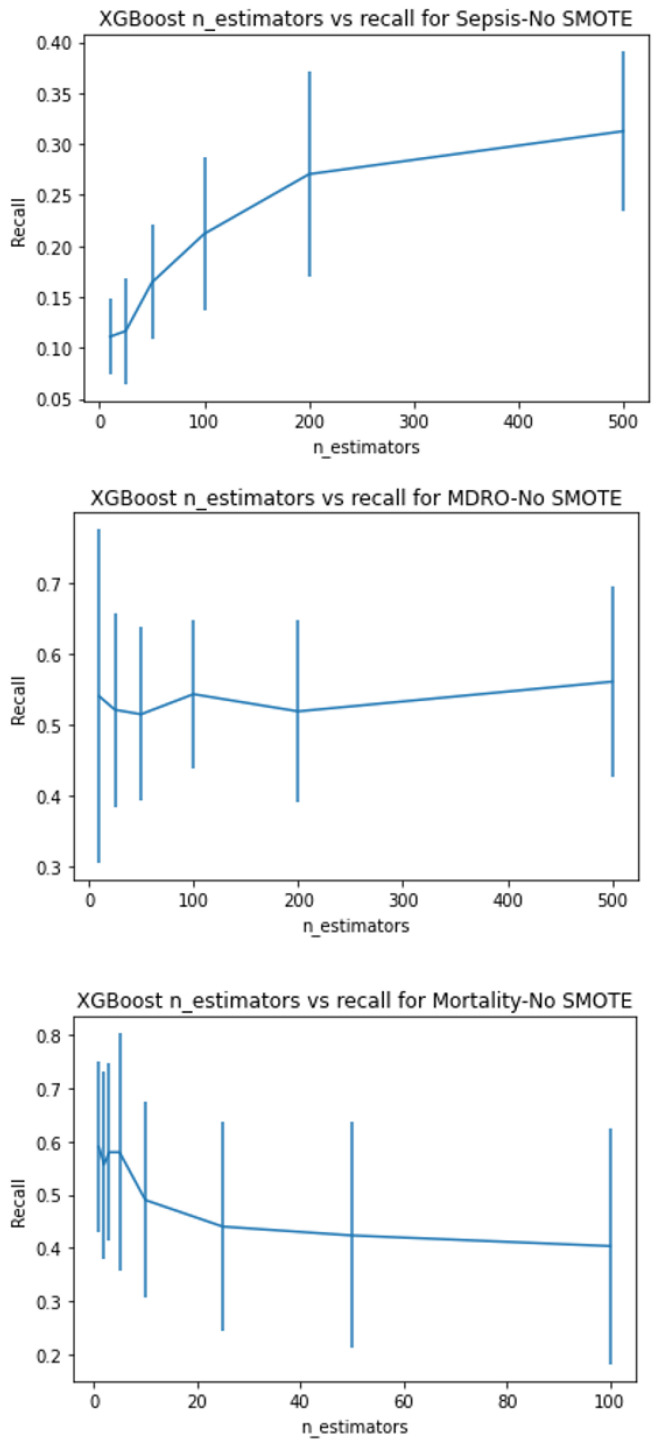
Shows the recall versus number of estimators for all three outcomes without data augmentation on the training set, plotted using XGboost as the base model.

**Figure 6:**
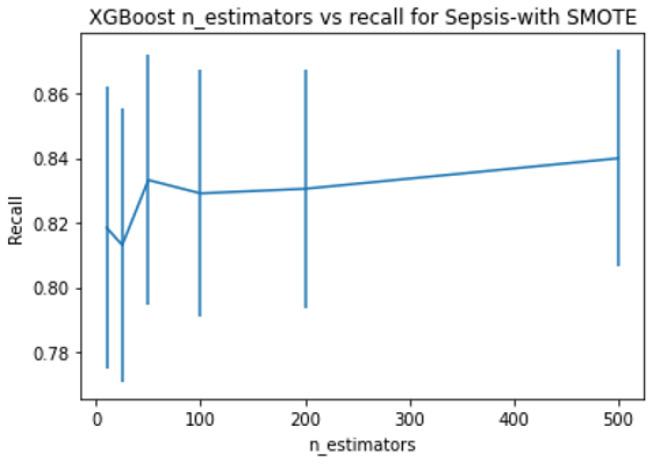

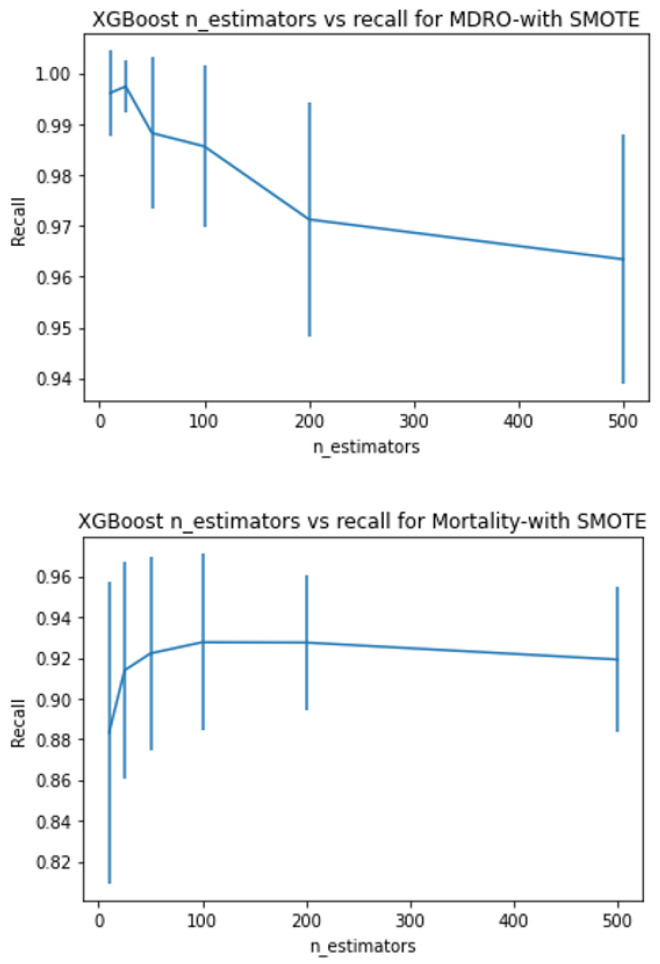
Shows the recall versus number of estimators for all three outcomes with data augmentation on the training set, plotted using XGboost as the base model.

XGboost has several tree-specific parameters and boosting parameters to limit overfitting. See Table S2 and the associated explanation in the supplementary file for more details about hyperparameter tuning. With hyperparameter tuning, the best model is chosen using 5-fold stratified cross-validation. The parameter range for all three search methods was kept the same. As random search looks for optimal values in a parameter space rather than on grids, the former is more likely to figure out the optimal parameter. However, both methods are prone to the curse of dimensionality and are computationally costly for a large number of variables. Hence, in order to compromise high computational requirements, yet arrive at the best, we tuned some of the parameters singly and then performed a combination search around the optimal value results. We checked the effect of the learning rate and the number of estimators for predicting sepsis, MDRO, and mortality by keeping other parameters at default. Figure 3, Figure 4, Figure 5 and Figure 6 show the recall versus learning rate and the number of estimators for all three outcomes in the ranges η=[0.01,0.05,0.1,0.2,0.3,0.4,0.5,0.6,1]n_estimators = [10, 25, 50, 100, 200, 500], respectively. Using 5-fold stratified CV, a subrange around the best values was identified and used in the case of grid, random, and hyperopt search modules for further tuning along with other parameters. Because the individual best parameters may not be the best when considered together with other hyperparameters. The results of the final parameters of XGboost obtained using grid, random, and hyperopt searches are given in Table S1 in the supplementary file. We used GridSearchCV and RandomizedSearchCV functions in Scikit-learn’s model_selection package and Hyperopt in the Bayesian optimization library. The simulations reported in this study were executed using Google Colab (web IDE (Integrated Development Environment)) with Colab notebook’s default language as Python on an Intel-i7 CPU.

## 3. Results

**Figure 7:**
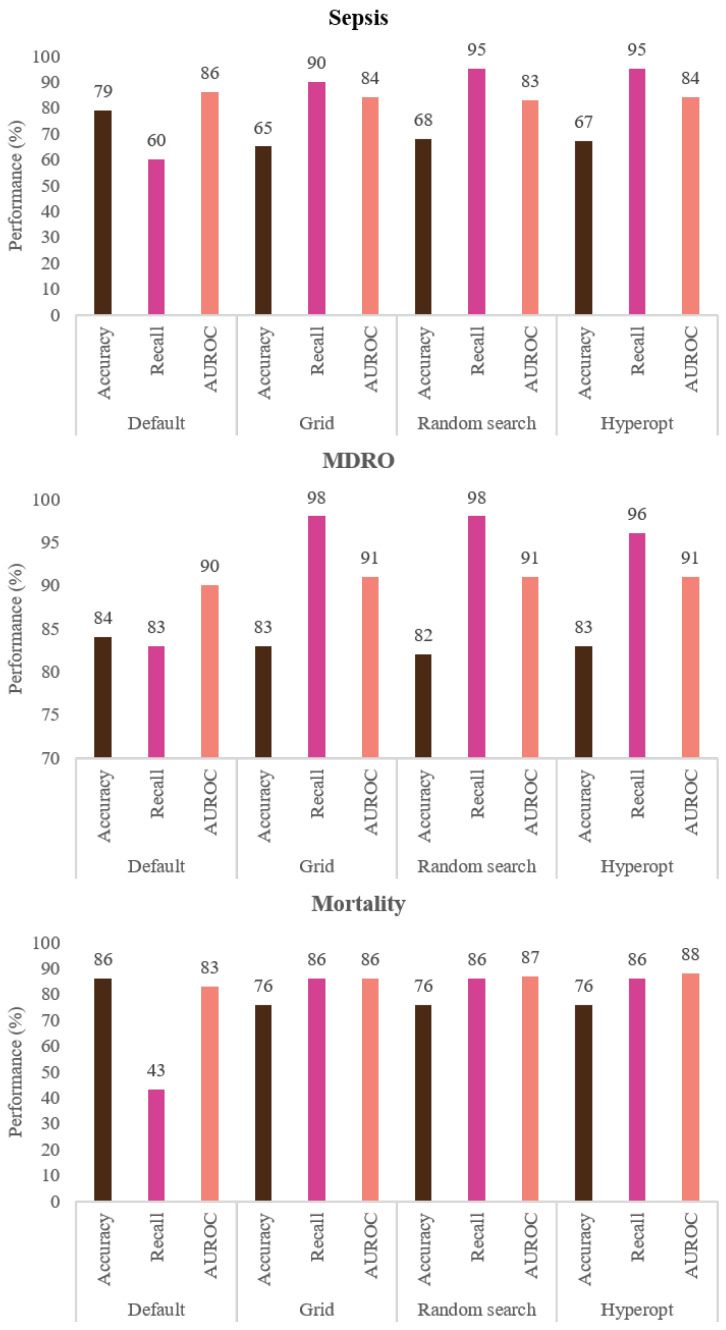
Shows the performance of the (1) default XGboost model before hyperparameter tuning, best model resulted from (2) grid search, (3) random search, and (4) Bayesian optimization (hyperopt) for each endpoint, obtained by using 5-fold stratified cross-validation with model scoring set to “recall”. Parameter n_iter=100 was set for random search.

Once the best model parameters (hyperparameters) are fixed in each case, as shown in Figure 1, the model is re-fitted using the training set, and the final model evaluation is conducted on the unseen test set. Figure 7 shows the performance of the default XGboost model versus the final best models for sepsis, MDRO, and mortality obtained via 5-fold stratified cross-validation using grid search, random search, and Bayesian optimization (hyperopt). The AUC curve of the XGboost model tuned using grid search, random search, and Bayesian optimization (hyperopt) for each endpoint is given in Figures S3–S5 in the supplementary file.

The sensitivity or recall (true positive/(true positive + false negative)) values obtained are 60%, 90%, 95%, and 95% for sepsis, 83%, 98%, 98%, and 96% for MDRO, and 43%, 86%, 86%, 86% for mortality when default, grid search, random search, and hyperopt-based XGboost models are used, respectively. Compared to the default model, considerable improvement in performance is achieved in terms of desirable performance metrics (i.e., recall in our case) by using appropriate model scoring metrics while conducting hyperparameter tuning. The value of AUC is slightly improved for MDRO and mortality. However, the increase in true positive detection and decrease in false negative is achieved at the cost of a slightly increased number of false positives, and hence reduced accuracy. As shown in Figure 3, Figure 4, Figure 5, Figure 6 and Figure 7, even though the recall of the XGboost model before hyperparameter tuning is less in the case of sepsis, MDRO, and mortality prediction, the recall has improved with all three types of parameters searching. Comparatively, hyperopt shows robust performance with less variance.

## 4. Discussion

The initial analysis of the study cohort revealed that sepsis was most prevalent in LYM (58/216 (27%)) followed by MDS (6/27 (22%)). No significant difference was noted in the presence of MDRO in AML (124/640 (20%)), LYM (43/216 (20%)), and MDS (6/27 (22%). Mortality within 30 days of FNE was similar in LYM (22/114 (20%)) and MDS (3/15 (20%)) while lesser in AML (34/266 (13%)) and ALL (7/118 (6%)) patients. Statistical inference analysis is important to understand the relationship between data features and can contribute toward building a predictive model. In this paper, we have used correlation analysis to understand the relationship between predictors and outcomes (Figures S1 and S2). Features such as line-related bloodstream infection, presence of polymicrobial infections (BSI-polymicrobial), and chest infection are the most correlated variables to the outcome sepsis. In the case of MDRO, along with line-related infections and chest infection, UTI, colitis, and skin infection showed a good correlation. Similarly, septic shock and chest infection showed the highest correlation to mortality. On par with the correlation analysis, the feature selection methods have also identified variables with good correlation with the respective outcome as the best features for prediction (Table 2, Table 3 and Table 4, Table S1 and Figures S1, S2, S6–S8). While statistical inference methods allow the evaluation of the relationship between the input features (predictors) and output features (clinical outcome), a trained ML model can be used to predict the occurrence of an event (outcome) ahead of its occurrence and hence is a promising tool for clinical decision-making.

In this paper, we showed that by amalgamating data augmentation techniques (SMOTE) along with setting specific scoring methods in hyperparameter tuning, considerable improvement can be achieved in the positive prediction quality of ML models. Even though we level up the number of positive (event or minority class) samples in the training set using SMOTE, the test set is untouched and contains no generated data. As shown in Figure 2, there is considerable improvement in recall in the case of SMOTE compared to the bootstrap method. Similarly, for all three outcomes, there is around a 50% reduction in the recall achievable with no data augmentation compared to with data augmentation (Figure 3, Figure 4, Figure 5 and Figure 6). This clearly shows the usefulness of SMOTE for improving the learnability of the model.

**Figure 8:**
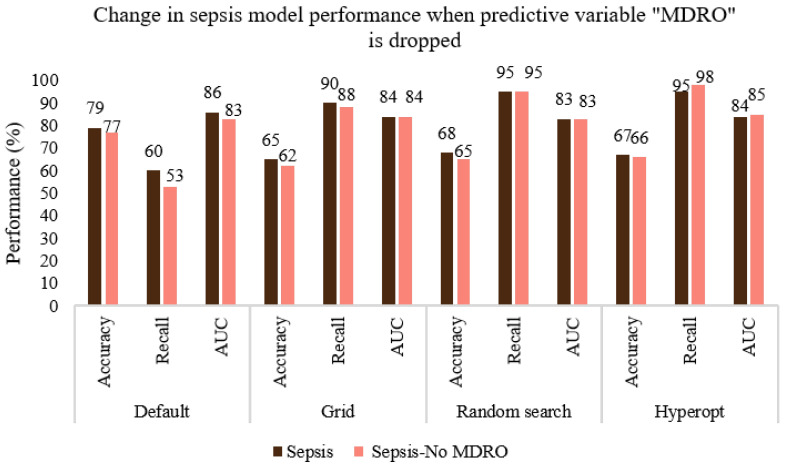
Comparing the performance of the default, grid search, random search, and Bayesian optimization (hyperopt) models when the predictive variable MDRO is removed from the feature set to predict sepsis.

**Figure 9:**
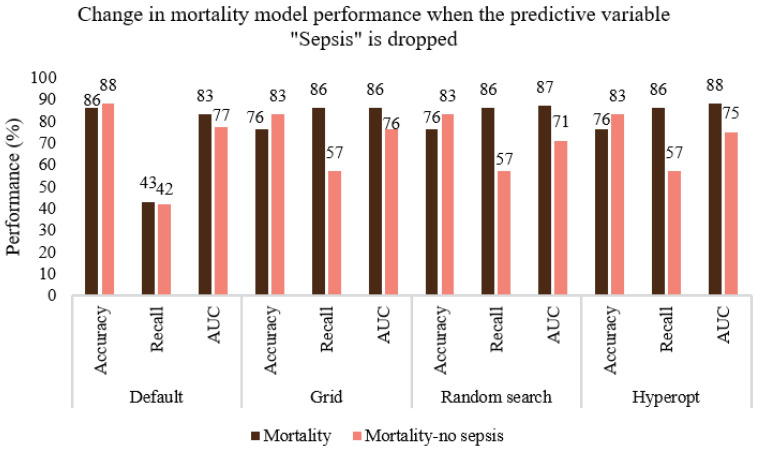
Comparing the performance of the default, grid search, random search, and Bayesian optimization (hyperopt) models when the predictive variable sepsis is removed from the feature set to predict mortality.

Considering the 12 final features used for the prediction of sepsis given in Table 2, obtaining the independent variable MDRO is relatively time-consuming and requires additional culture sensitivity experiments. Hence, we considered omitting the variable “MDRO” while predicting sepsis. As shown in Figure 8, AUC values remained more or less the same when the predictive variable “MDRO (yes/no)” was removed from the feature set to predict sepsis and an average drop of only 1.5% was noticed in the recall (sensitivity) value. The hyperopt search resulted in a better model even without MDRO and showed improved sensitivity (98%) and AUC (85%). We also noted that the specificity (true negative/(true negative +false positive)) values for default and hyperopt-tuned XGboost models were 84% and 62%, respectively. This indicates that when the model is fine-tuned to improve sensitivity (98%), its specificity decreases (62%). Similarly, Figure 9 shows the change in the performance of the predictive model for mortality when the independent variable “sepsis” is removed from the feature set. An average drop of 22% and 11% is noticed in the case of recall and AUC, respectively. Note that the sensitivity and specificity values of the hyperopt-tuned model for mortality prediction were 59% and 92%, respectively; this disparity is due to the rareness of the event (Table 4), with n = 66, only 15% of patients in the mortal group. Hence the model has less opportunity to learn positive cases. Based on the simulations conducted, the hyperopt search method is more robust (Figure 8), and it can converge to a global optimal value of parameters if a wider range of parameters is used for all hyperparameters.

Similar to the scenarios shown in Figure 8 and Figure 9, in the case of MDRO prediction, we omitted variables such as “line-related (yes/no)” and “BSI-polymicrobial” from the predictive variables and studied model performance. The accuracy, recall, and AUC values using the hyperopt model dropped down to 65%, 59%, and 63%, respectively, wherein the model identified 27/26 MDRO and 125/188 non-MDRO patients.

The main highlights of this paper are threefold, the (1) use of a data augmentation method to address disproportionate class issue, (2) tuning of XGboost’s hyperparameters to improve positive prediction, and (3) the introduction of a new set of feature sets for the prediction of sepsis, MDRO, and mortality in hematological patients with FN. The proposed models support clinical decision-making and allow medical staff to receive a quick and reliable second opinion to ascertain their intuition, relieve burden, improve preparedness for better management of MDRO lead nosocomial infection spread, and spare time for other critical tasks involved in patient management wherein human expertise is inevitable. The limitations of this study include class bias, as 400/513 (77.97%) of the patients are males. Recent research points out that patients with FN are frequently hospitalized despite being in a low-risk category as per clinical guidelines, which escalates health-care burden [26]. Hence, strategies to identify both low-risk and high-risk patients are desirable.

## 5. Conclusions

Hematologic cancer patients with FN should be screened for MDRO, septic shock, and mortality susceptibility to improve the standard of care and ensure vigilant surveillance of high-risk patients. While adopting artificial intelligence-driven strategies for identifying such clinical endpoints, class imbalance in the dataset due to disproportionate classes is a common issue that limits the learning opportunity of a model. This will lead to the development of a poorly performing predictive model, especially in predicting minority classes. Appropriate use of data augmentation methods, such as SMOTE and tuning the model parameters based on metrics that evaluate the positive predictive value of the model, can considerably boost the performance of the model. Our experiments show that, compared to the study without data augmentation, the one with SMOTE oversampling resulted in a 50% increase in recall. This paper also introduces a new set of features available from medical records of patients upon admission to reliably predict sepsis, MDRO, and mortality. The investigation of possible performance improvement of any of the proposed strategies are used with a few more clinically significant and easily available predictive features is underway. Futuristic healthcare strategies involving artificial intelligence-driven forecasting can help to improve cancer care, reduce mortality, and take proactive measures to devise a mitigation strategy against the potential spread of MDROs in a healthcare setting.

## Figures and Tables

**Table 1:** Characteristics of potential features in the dataset, F: fungi, GP: gram-positive, GN: gram-negative, MENA: Middle East and North Africa.

Features (n = 1166)	Values
Age (mean ± std. dev)	40.30±14.51
**Infection**	
Bloodstream infection, n (%)	427 (36.62%)
Chest infection, n (%)	260 (22.22%)
Sinus infection, n (%)	11 (0.94%)
Skin infection, n (%)	78 (6.68%)
Colitis, n (%)	86 (7.37%)
Urinary tract infection, n (%)	79 (6.8%)
**Gender**	
Male, n (%)	925 (79.33%)
Female, n (%)	241 (20.66%)
**Diagnostics category**	
ALL, n (%)	283 (24.27%)
AML, n (%)	640 (54.88%)
LYM, n (%)	213 (18.26%)
MDS, n (%)	27 (2.31%)
**Type of microorganism in BSI**	
F/GN,F/GP,GP,F,n(%)	17 (3.98%)
GN, n (%)	337 (78.92%)
GN, GP, n (%)	19 (4.45%)
GP, n (%)	54 (12.65%)
**Region**	
R1- South Asia, n (%)	497 (42.62%)
R2- MENA, n (%)	424 (36.36%)
R3- East Pacific, n (%)	166 (14.23%)
R4- Sub-Sahara Africa, n (%)	55 (4.71%)
R5- Others, n (%)	24 (2.05%)
**Treatment phase**	
Pretreatment, n (%)	166 (14.23%)
Induction for remission, n (%)	323 (27.70%)
Post induction, n (%)	507 (43.48%)
Salvage therapy, n (%)	51 (4.37%)
Palliative, n (%)	119 (10.21%)
**Disease status**	
Complete/partial response, n (%)	548 (47.00%)
Refractory/Relapse, n (%)	194 (16.64%)
Others, n (%)	424 (36.36%)
**Outcome**	
Sepsis, n (%)	229 (19.64%)
MDRO, n (%)	215 (18.43%)
Mortality, n (%)	66 (12.86%)

**Table 2:** Distribution of features in the sepsis and non-sepsis groups, number of FNEs, n = 1166. Along with the features listed below, other multi-category features, such as region of origin, diagnostic category, treatment phase, disease status, and type of microorganism in the bloodstream, were used to predict sepsis. A total of 12 variables were used in the model to predict sepsis.

Features	Sepsis Group (n = 229)	Non-Sepsis Group (n = 937)
Age	42.16 ± 15.60	39.8 ± 14.19
Sex (male)	174 (75.98%)	751 (80.14%)
Line-related	86 (37.55%)	112 (11.95%)
BSI-polymicrobial	31 (13.54%)	32 (3.41%)
Chest infection	98 (42.79%)	162 (17.29%)
UTI	31 (13.54%)	48 (5.1%)
MDRO	96 (41.92%)	119 (12.70%)

**Table 3:** Distribution of features in the MDRO and non-MDRO groups, number of FNEs, n = 1166. Along with the features listed below, other multi-category features, such as region of origin, diagnostic category, treatment phase, disease status, and type of microorganism in the bloodstream, were used to predict MDRO. A total of 13 variables were used in the model to predict MDRO.

Features	MDRO Group (n = 215)	Non-MDRO Group (n = 951)
Age	40.83 ± 14.68	40.17 ± 14.46
Sex (male)	163 (75.81%)	762 (80.12%)
Line-related	113 (52.56%)	85 (8.93%)
BSI-polymicrobial	43 (20%)	20 (2.1%)
Chest infection	65 (30.23%)	195 (20.50%)
Colitis	32 (14.88%)	54 (5.67%)
UTI	23 (10.69%)	56 (5.89%)
Skin infection	30 (13.95%)	48 (5.04%)

**Table 4:** Distribution of features in the mortal and non-mortal groups, number of patients, n = 513. Along with the features listed below, other multi-category features, such as diagnostic category, treatment phase, disease status, and type of microorganism in the bloodstream, were used to predict mortality. A total of 8 variables were used in the model to predict mortality.

Features	Mortal Group (n = 66)	Non-Mortal Group (n = 447)
Age	42.28 ± 16.60	40.08 ± 14.59
Sex (male)	53 (80.30%)	343 (76.73%)
Chest infection	40 (60.60%)	100 (22.37%)
Sepsis	55 (83.33%)	51 (11.41%)

## Data Availability

Data are available from E.A., upon reasonable request.

## Supplementary Materials

The following supporting information can be downloaded at: https://www.mdpi.com/article/10.3390/ijerph20010526/s1.

## Abbreviations

The following abbreviations are used in this manuscript:

NCCCR	The National Center for Cancer Care and Research
FN	Febrile Neutropenia
AUC	Area Under the Curve
MDRO	Multi-Drug-Resistant Organism
SDG	Sustainable Development Goals
WHO	World Health Organization
ALL	Acute Lymphoid Leukemia
AML	Acute Myeloid Leukemia
LYM	Lymphoma
MDS	Myelodysplastic Syndromes
BSI	Bloodstream Infection
UTI	Urinary Tract Infection
ML	Machine Learning
AI	Artificial Intelligence
MASCC	Multinational Association for Supportive Care in Cancer
ANN	Artificial Neural Network
GCSF	Granulocyte Colony-Stimulating Factor
CBC	Complete Blood Count
CRP	C-reactive protein
LSVM	Linear support vector machine
LR	ridge-Logistic Regression
GBT	Gradient-Boosting Tree
GN	Gram-negative
GP	Gram-positive
SMOTE	Synthetic Minority Oversampling Technique
BO	Bayesian Optimization
TPE	Tree Parzen Estimator
QBRI	Qatar Biomedical Research Institute
HMC	Hamad Medical Corporation
