# Supplementary material for: Prediction of Multiple Clinical Complications in Cancer Patients to Ensure Hospital Preparedness and Improved Cancer Care
Source: Int J Environ Res Public Health. 2022 Dec 28;20(1):526. doi: 10.3390/ijerph20010526 (PMC9819091; doi:10.3390/ijerph20010526)
Supplement: Supplementary file 1 [file ijerph-20-00526-s001.zip › ijerph-1962412-supplementary.pdf]

International Journal of
Environmental Research and Public Health

MDPI

Article

Prediction of Multiple Clinical Complications in Cancer Patients to Ensure Hospital Preparedness and Improved Cancer Care

Regina Padmanabhan [1,*], Adel Elomri [1], Ruba Yasin Taha [2], Halima El Omri [2], Hesham Elsabah [2] and Abdelfatteh El Omri [3]

1 Division of Engineering Management and Decision Sciences, College of Science and Engineering, Hamad Bin Khalifa University, Qatar Foundation, Doha 34110, Qatar
2 Department of Hematology and Bone Marrow Transplant, National Center for Cancer Care and Research, Hamad Medical Corporation, Doha 3050, Qatar
3 Surgical Research Section, Department of Surgery, Hamad Medical Corporation, Doha 3050, Qatar
* Correspondence: rpadmanabhan@hbku.edu.qa

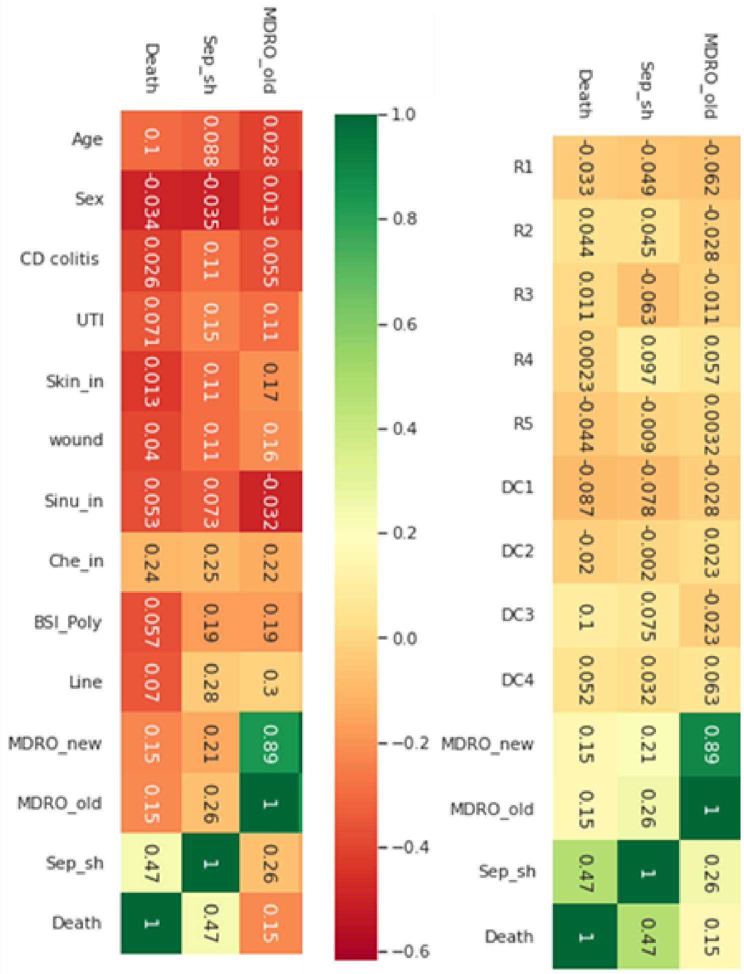

Figure S1. Heatmap showing the correlation of features such as age, sex, CD colitis, urine tract infection, wounds, sinus infection, chest infection, BSI polymicrobial (yes/no), line-related infection (yes/no), region 1 (R1—south Asia), region 2 (R2—MENA), region 3 (R3—east Asia Pacific), region 4 (R4—sub-Sahara-Africa), region 5 (R5—others, including north America, Europe), disease category 1 (ALL), DC2 (AML), DC3 (LYM), DC4 (MDS), with output variables MDRO, septic shock, and death in order.

check for updates

Citation: Padmanabhan, R.; Elomri, A.; Taha, R.Y.; El Omri, H.; Elsabah, H.; El Omri, A. Prediction of Multiple Clinical Complications in Cancer Patients to Ensure Hospital Preparedness and Improved Cancer Care. *Int. J. Environ. Res. Public Health* **2023**, *20*, 526. https://doi.org/10.3390/ijerph20010526

Academic Editor: Zahra Mojtahedi and Shirin Farjadian

Received: 25 September 2022
Revised: 22 November 2022
Accepted: 4 December 2022
Published: 28 December 2022

Copyright: © 2022 by the authors. Licensee MDPI, Basel, Switzerland. This article is an open access article distributed under the terms and conditions of the Creative Commons Attribution (CC BY) license (https://creativecommons.org/licenses/by/4.0/).

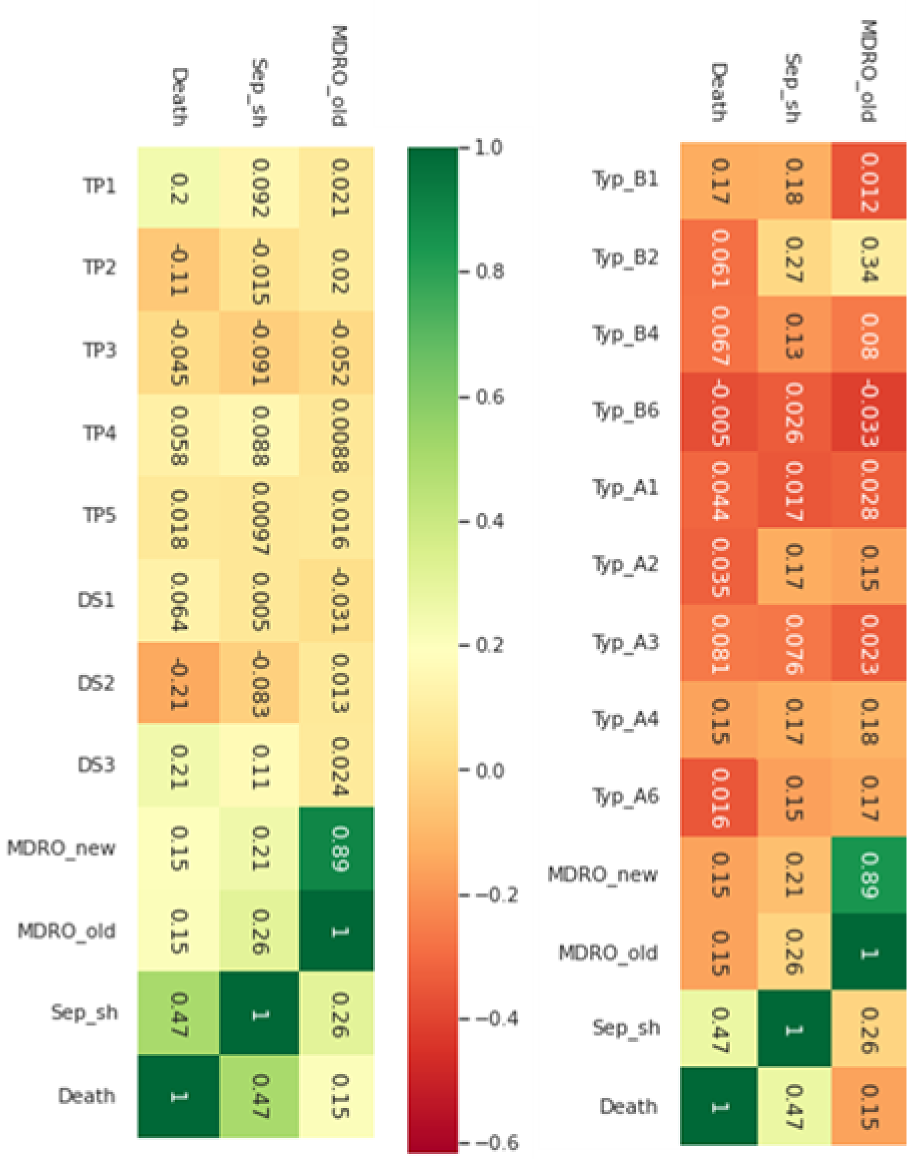

Figure S2. Heatmap showing the correlation of features such as such as treatment phase 1 (TP1) (palliative), TP2 (induction consolidation to sustain remission), TP3 (induction to achieve remission), TP4 (Salvation therapy), TP5 (pretreatment), disease status 1 (DS1) (not applicable), DS2 (complete/partial response), DS3 (refractory/relapse), BSI (Typ_B1) (Gram-negative), Typ_B2 (Gram-positive), Typ_B4 (Fungal/Gram-negative/Gram-positive), Typ_B6 (Gram-negative/Gram-positive), type of causative organism in all 6 infections (Typ_A1) (Gram-negative), Typ_A2 (Gram-positive), Typ_A4 (Fungal/Gram-negative/Gram-positive), Typ_A6 (Gram-negative/Gram-positive), MDRO, septic shock, and death in order.

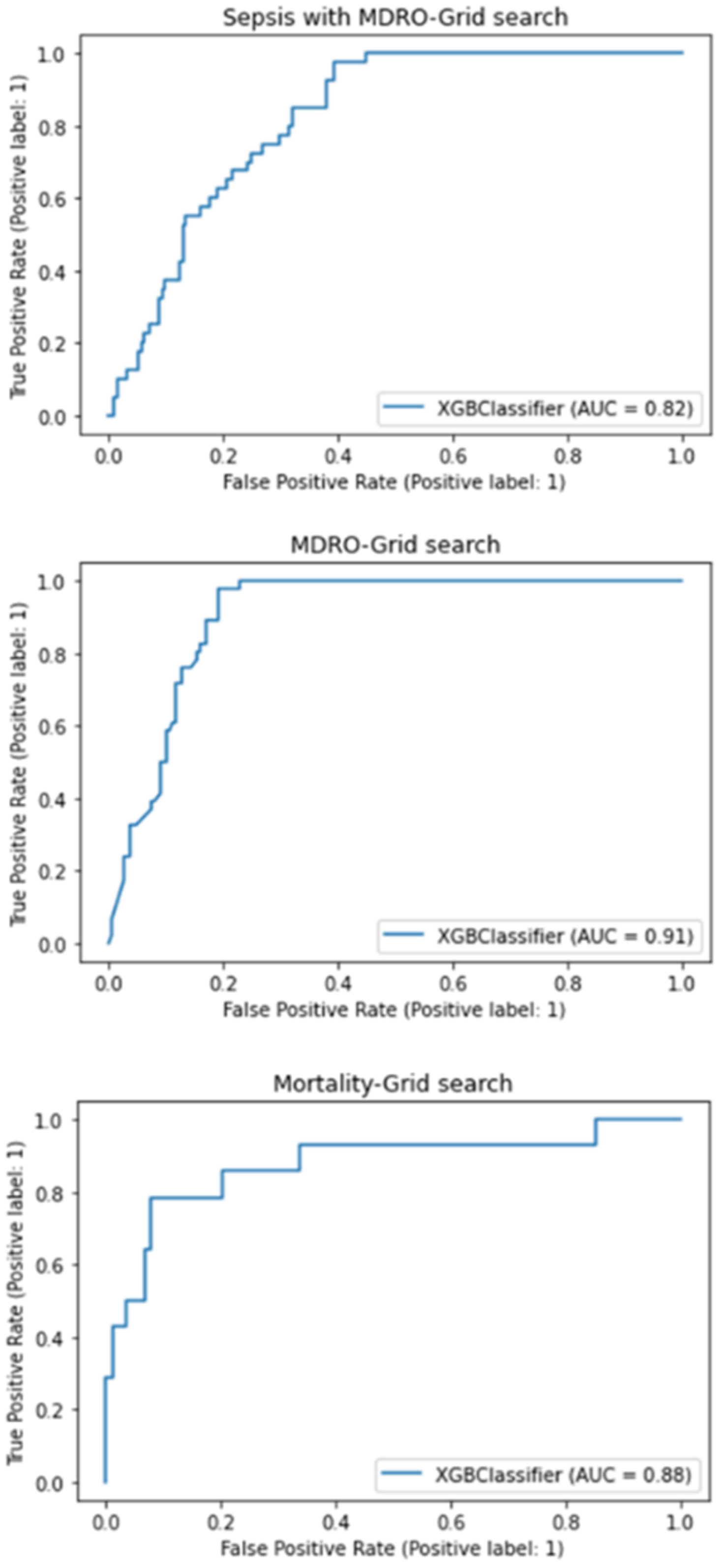

Figure S3. AUC curves of the XGboost model tuned using grid search for each endpoint.

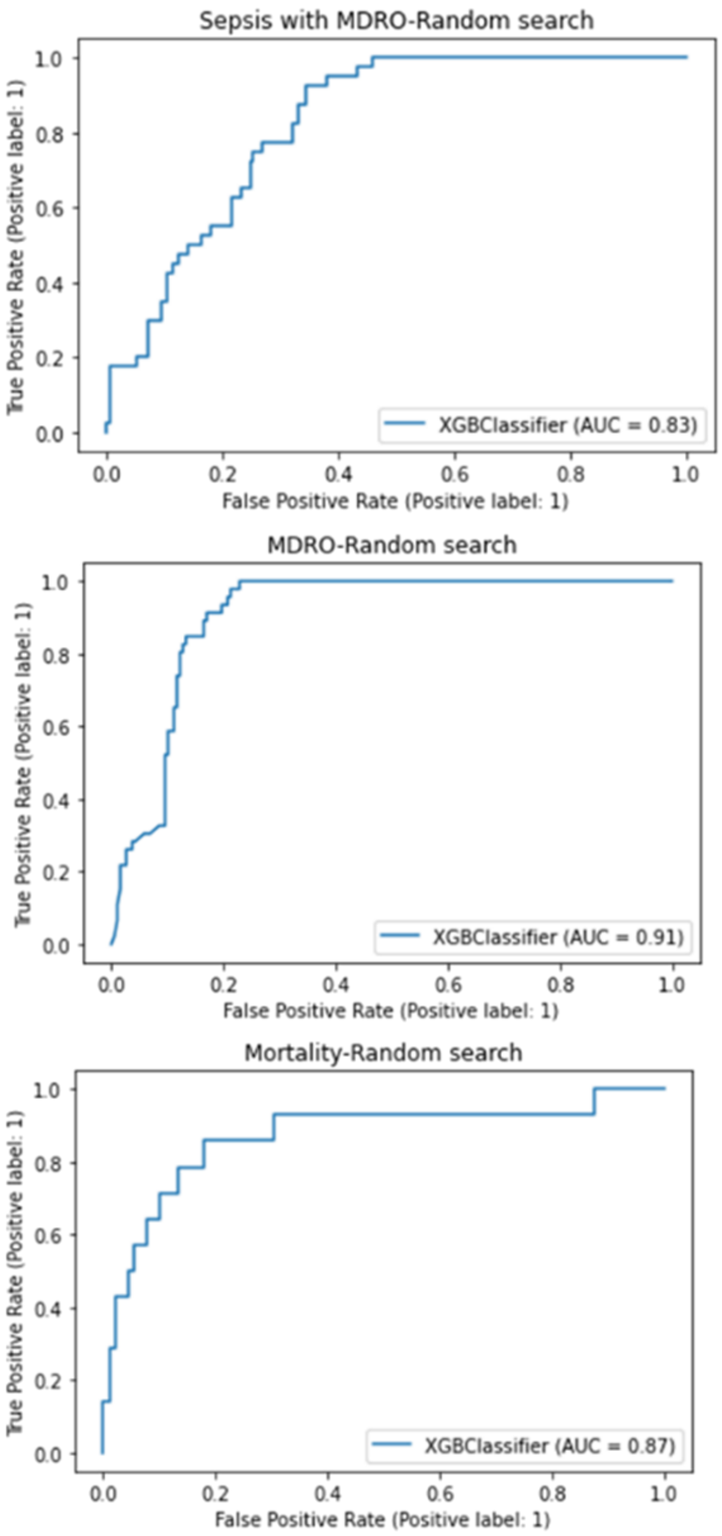

Figure S4. AUC curves of the XGboost model tuned using random search for each endpoint.

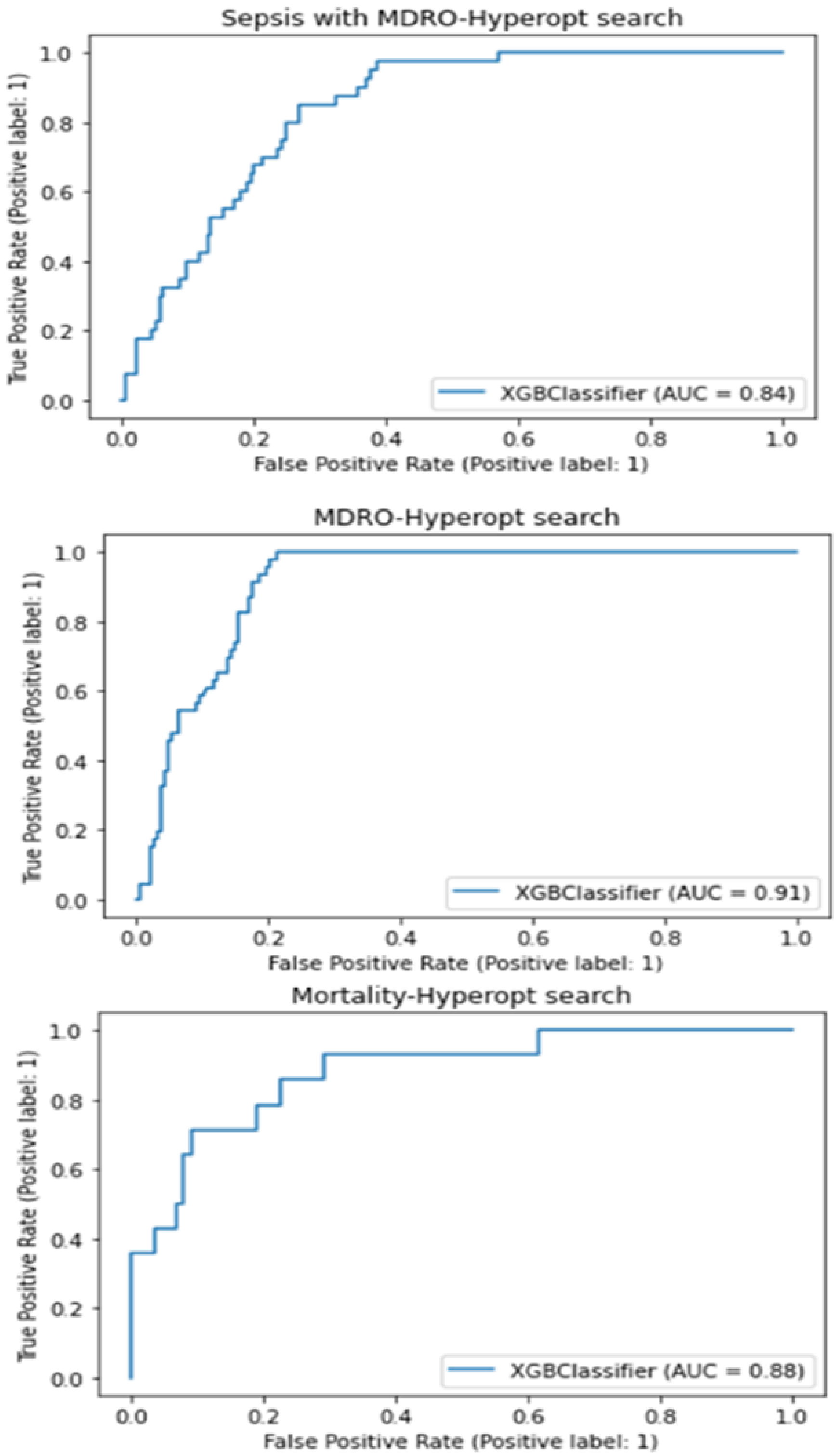

Figure S5. AUC curve of the XGboost model tuned using hyperopt search for each endpoint.

Table S1. Important features derived using the logistic regression classifier as the base model.

Outcome	Feature
Sepsis	'Skin_in', 'Che_in', 'Line', 'R4', 'DC4', 'TP3', 'TP4', 'DS2', 'Typ_B1', 'Typ_B2', 'Typ_B4', 'Typ_A4', 'Typ_A6', 'MDRO_new', 'MDRO_old'
MDRO	'Age', 'UTI', 'Skin_in', 'Che_in', 'Line', 'R3', 'R5', 'DC4', 'TP4', 'TP5', 'Typ_B2', 'Typ_A1', 'Typ_A3', 'Typ_A4', 'Typ_A6'
Mortality	'Sex', 'Sinu_in', 'Che_in', 'R3', 'R4', 'R5', 'DC3', 'TP1', 'TP4', 'DS2', 'DS3', 'Typ_A2', 'Typ_A3', 'Typ_A6', 'Sep_sh'

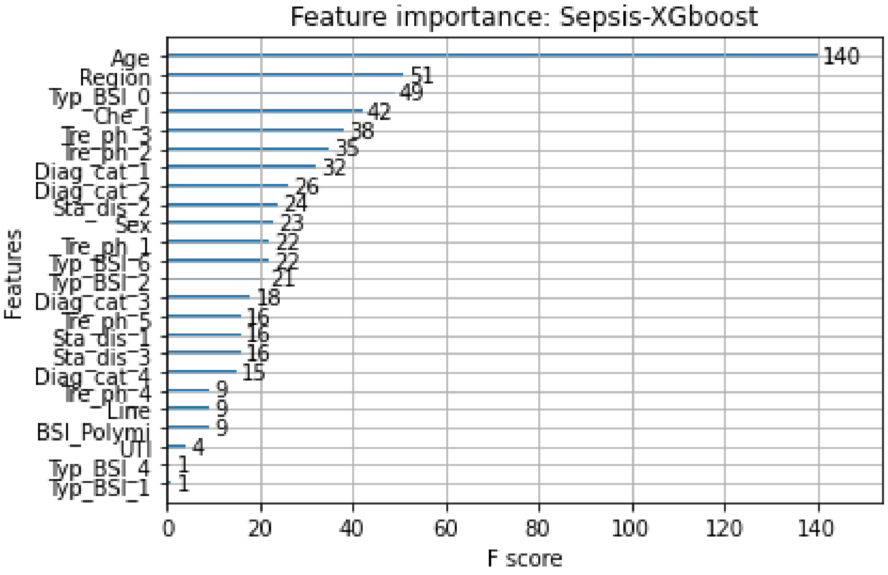

Figure S6. Important features to predict sepsis, derived using XGboost as the base model.

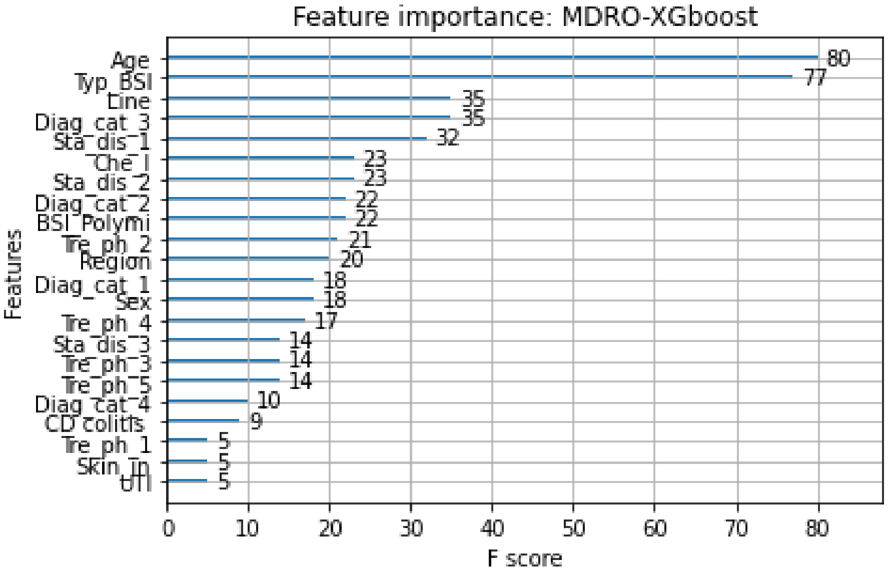

Figure S7. Important features to predict MDRO, derived using XGboost as the base model.

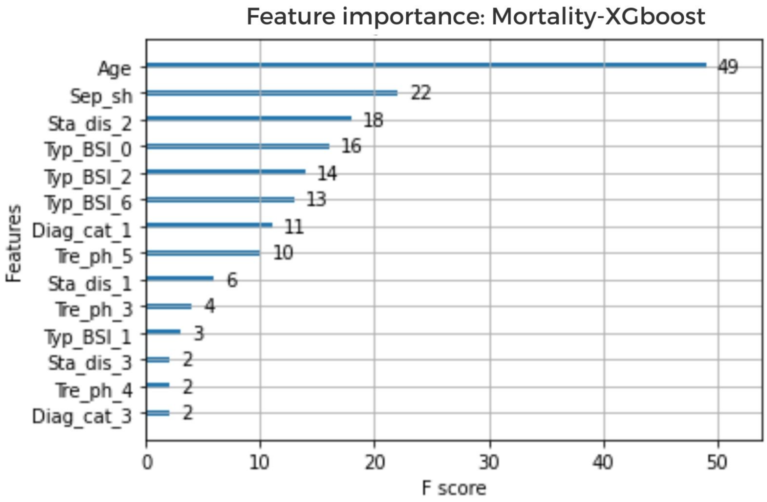

Figure S8. Important features to predict mortality, derived using XGboost as the base model.

Table S2. Results of final parameters of XGboost obtained using grid, random, and hyperopt search. The best model is chosen using 5-fold stratified cross-validation.

**Xgboost parameters**	**Grid (Sepsis, MDRO, Mortality)**	**Random Search (Sepsis, MDRO, Mortality)**	**Hyperopt (Sepsis, MDRO, Mortality)**
col_sample_by_tree	0.5, 0.8, 0.5	0.8, 0.8, 0.5	1, 1, 0.5
gamma	0, 0, 0	0.25, 0.01, 0.01	0.074, 0.117, 0.003
learning_rate	0.05, 0.001, 0.1	0.05, 0.01, 0.01	0.174, 0.008, 0.021
max_depth	7, 7, 7	7, 7, 8	8, 8, 10
n_estimator	25,10, 25	25, 10, 100	25, 25, 25
reg_lambda	1, 1, 1	1, 1, 1	0.097, 0.389, 0.007
scale_pos_weight	7, 3, 7	7, 3, 7	5.374, 1.004, 5.225
Subsample_space	0.5, 0.8, 0.5	0.8, 0.8, 0.5	1, 1, 1

We tuned the *tree-specific parameters*, such as maximum depth (*max_depth*) of the tree and gamma (γ), to control over-fitting, as higher depth makes the model learn specific relations pertaining to the training set, parameter γ is adjusted according to the loss reduction threshold that decides the partition of a leaf node, which is a split and is recommended only if it results in a better (less) loss function. Similarly, for *boosting parameters*, such as *learning_rate* (η), which determines the rate and magnitude of updates of estimates, lower values lead to a robust and more generalizable model at the cost of accommodating a higher number of trees, which demands more computational power. *n_estimators* decides the number of sequential trees, the choice of which is robust at higher numbesr, but beyond a point leads to overfitting; more trees also means more computation. For the boosting parameter *subsample*, which determines the fraction of observations to be selected for each tree, values close to 1 are recommended to derive a robust model, tuning is used to find an optimal value between 0.5 and 1, to find the lowest subsample with the least variance. The parameter *scale_pos_weight* of XGboost can handle data imbalances, and it roughly adds an effect similar to oversampling of the dataset. *scale_pos_weight* is also in the boosting category, which scales the gradient for positive (minority) classes. Similarly, miscellaneous parameters, such as random number seed (*random_state*), are set to an integer value for reproducibility. Parameters α and λ are used to set L1 and L2 regularization parameters, respectively. Tree growth policy is also critical, we can choose depth wise or loss guide to decide how further nodes are added to the tree. Out of the objective function options for binary classification in XGboost, we used binary:logistic as objective function of XGboost.
